# Breast Cancer Brain Metastasis: A Comprehensive Review

**DOI:** 10.1200/OP.23.00794

**Published:** 2024-05-15

**Authors:** Akshara S. Raghavendra, Nuhad K. Ibrahim

**Affiliations:** ^1^ Department of Breast Medical Oncology, The University of Texas MD Anderson Cancer Center, Houston, TX

## Abstract

The mechanisms underlying breast cancer brain metastasis (BCBM) development are complex, and its clinical presentation varies depending on the number, location, and size of brain metastases. Common symptoms include headache, neurologic deficits, and seizures. Diagnosis of BCBM typically relies on neuroimaging techniques, such as magnetic resonance imaging and computed tomography scans. Local therapies, such as surgery and stereotactic radiosurgery, can be used to control tumor growth and relieve symptoms. Whole-brain radiotherapy has been a mainstay of treatment for BCBM, but its use has been associated with cognitive decline. Systemic therapy with chemotherapy and targeted agents plays an increasingly important role in the management of BCBM. Novel agents, such as human epidermal growth factor receptor 2 (HER2)–targeted therapies and tyrosine kinase inhibitors, have shown promising results in improving survival for patients with HER2-positive and triple-negative BCBM. This comprehensive review synthesizes current knowledge, clinical insights, and evolving paradigms to provide a robust understanding and roadmap for optimizing the diagnosis and management of BCBM.

## Teaser

Improved Management of BCBM through Multi-Disciplinary Approaches, Leads to Enhanced Patient Outcomes

## INTRODUCTION

Brain metastases (BMs) occur in 20%-40% of patients with breast cancer during the course of their disease.^1^ BMs from solid tumors contribute significantly to morbidity and/or mortality, with about 200,000 patients—10% of all patients with cancer—diagnosed each year in the United States.^2^ Notably, Breast Cancer BMs (BCBM) represents second most common (30%) among all cancers.^3^ With advancements in diagnostic techniques and systemic disease treatment, the incidence of BMs is on the rise.^5^ Dissemination of breast cancer to the CNS occurs in 15%-30% of patients with invasive breast cancer, resulting in worse outcomes.^6^

BMs are more common in certain breast cancer subtypes, such as human epidermal growth factor receptor 2 (HER2)–positive breast cancer, which has a higher incidence of BMs (35%-50%) but generally yields better survival outcomes compared with HER2-negative disease.^6,7^ Triple-negative breast cancer (TNBC) is associated with a high frequency (34%) of BMs and poor survival outcomes.^8,9^ TNBC is also associated with *gBRCA1* pathogenic mutations, which carry an elevated risk of brain-first recurrence.^10^ Compared with *gBRCA2* carriers, BM is more prevalent among patients carrying *gBRCA1* mutations. Notably, *gBRCA1* patients have a shorter median time to BM detection from the initial breast cancer diagnosis (2.4 years). Conversely, *gBRCA2* patients, who more often have hazard ratio (HR)-positive/HER2-negative tumors, had a longer time to BM detection (5 years). In addition, individuals with *gBRCA1* tumors tend to experience BMs at a younger age than do patients with *gBRCA2* tumors. Moreover, those with *gBRCA2* tumors often exhibit a higher occurrence of multiple brain lesions rather than solitary lesions although they generally have a longer overall survival (OS) rate, compared with *gBRCA1*^11^ patients. HR-positive patients account for 14% of breast cancer–related BMs.^12^ Moreover, inflammatory breast cancer (IBC) manifests a significant predilection toward BMs, which are reported in 13.2% of patients with IBC, signifying a notably elevated risk of developing BMs.^13^ The cumulative incidence rates of BMs at 2 years, 5 years, and 10 years were 9.8%, 15.8%, and 17.4% for patients with IBC. For patients without IBC, the corresponding rates were 6.5%, 10.1%, and 12.7%.^14^

## DIAGNOSIS

When clinically suspected, BMs can be confirmed by computed tomography appear as solitary or multiple mass lesions with variable surrounding vasogenic edema. However, magnetic resonance imaging (MRI) is frequently used because of its ability to show more details about the size, number, and distribution of metastases. The most commonly observed patterns in BMs are solid enhancement and rim enhancement, often accompanied by a central cystic nonenhancing region.^23^ On the other hand, the sensitivity of fluorodeoxyglucose positron emission tomography is also limited for small lesions, with a sensitivity of 27% compared with contrast-enhanced MRI for BMs.^24^

## PROGNOSTIC FACTORS

Several clinical features have been associated with an increased risk of developing BMs from breast cancer, including young age (younger than 50 years), metastatic involvement of four or more axillary lymph nodes, and high tumor grade.^25^

There are three distinct patterns of BM. In the first pattern, the brain is the initial or sole site of metastasis: Approximately 10% of individuals diagnosed with metastatic breast cancer will initially manifest with BMs, the median time to occurrence is approximately 12.8 months from the primary tumor diagnosis.^26^ The second pattern is characterized by bimodal CNS recurrence, including early BM recurrence, occurring within 1-2 years from the initial diagnosis of breast cancer,^28^ and third with delayed brain metastasis recurrence, taking place more than 4-5 years after the diagnosis of breast cancer.

Nomograms can estimate the likelihood of BMs on the basis of clinical and pathologic variables, allowing for personalized risk assessment and help enrich selection of high-risk patients in the design of prophylactic therapy trials.^14,29-31^ Younger age, higher tumor grade, a greater number of metastatic sites, involvement of the lungs, shorter disease-free survival, and negative hormone status can serve as indicators of increased risk of BMs in patients with metastatic cancer.^31-33^

The median OS for patients with breast cancer diagnosed with untreated BMs is 1 month,^37^ treatment with surgical excision, whole-brain radiotherapy (WBRT), or stereotactic radiosurgery (SRS) prolongs survival to 1-2 years, and OS varies significantly on the basis of molecular prognostic factors and cancer subtypes.^37^ The OS is around 14 months for TNBC, 18 months for HER2-positive breast cancer, and 34 months for luminal breast cancer after treatment of BMs.^1,38,39^ The cause of death is influenced by tumor subtype, with HER2-positive patients more likely to die of CNS progression and patients with TNBC more likely to die of extracranial metastatic disease.^40^

Sperduto et al^23,41^ introduced the breast cancer disease–specific graded prognostic assessment highlighting the effect of tumor subtype, Karnofsky performance status (KPS), and age on survival and its incorporation in predicting outcomes for patients with breast cancer and BMs (Table 1): the lower the score, the worse the prognosis. Other prognostic scoring systems developed to help choose the best treatment options include the Breast Recursive Partitioning Analysis Prognostic Index,^42,43^ the Simple Survival Score for Patients with BM from Breast Cancer,^44^ and the Modified Breast Graded Prognostic Assessment.^45,46^

**TABLE 1.** Graded Prognostic Assessment Index for Women With Breast Cancer and Brain Metastases

Factor	0.0	0.5	1.0	1.5	2.0
KPS	≤50	60	70-80	90-100	—
Genetic subtype	Basal	—	Luminal A	HER2	Luminal B
Age, years	≥60	<60	—	—	—

Abbreviations: HER2, human epidermal growth factor receptor-2; KPS, Karnofsky performance status.

## LOCAL MANAGEMENT OF BREAST CANCER BMS

Key factors to consider in the management of BMs from breast cancer include assessing their number and location, therefore distinguishing between single and oligometastatic (multifocal) metastases that may be candidates for surgical resection versus multicentric spread not amenable to surgery. Other factors include the size of the individual lesions, location, and possible neurologic impairment. Historically, WBRT was the standard of care in the 1970s, resulting in a median survival of 4-5 months.^47,48^

Surgical resection was introduced in the 1980s for patients with operable single or oligometastatic BMs. In the early 1990s, studies evaluated the effectiveness of surgical intervention in prolonging survival while improving neurologic function and overall performance status.^49^ In a randomized trial comparing surgery followed by WBRT versus WBRT alone in patients with a single BM, those who underwent surgery followed by radiotherapy had a reduced risk of recurrence at the original metastatic site (5 of 25 [20%] *v* 12 of 23 [52%]; *P* = .02), longer OS (median, 40 weeks *v* 15 weeks; *P* = .01), and a longer period before experiencing a decline in performance status.^50^ A European trial confirmed the effectiveness of combining WBRT with surgery for single BMs compared with WBRT alone; the median OS for the two groups was 10 months and 6 months, respectively (*P* = .04).^51^ Both these studies (N = 63), in which most primary lesions were lung cancer (n = 33, 52%) or breast cancer (n = 12, 19%), showed that patients who undergo surgery have advantages over those who do not, including quick recovery of symptoms, particularly among those with good performance status and stable extracranial disease. However, 13% of patients who undergo surgery for BMs experience major complications such as neurologic worsening, meningitis including leptomeningeal carcinomatosis (LMD) secondary to CSF seeding, stroke, seizure, focal neurologic deficits, hemorrhage, tumor seeding, infection, worsened pulmonary function, and long recovery times. Because of its high morbidity and mortality, surgery is not recommended for patients who have a poor prognosis or rapidly progressive disease.^52^

### SRS

SRS is an alternative treatment option for oligometastatic BMs. SRS delivers accurate and concentrated high-dose radiation to specific areas, effectively eliminating macroscopic disease while minimizing damage to the surrounding healthy tissues by using multiple well-collimated beams of radiation to target specific areas of the brain.^53^ SRS delivered a 10 times higher dose of radiation compared with WBRT in a single- or multiple-beam fraction, allowing for a more immediate dose falloff around the targeted lesion, thereby minimizing radiation exposure to surrounding healthy tissues.^53^ Unlike WBRT, SRS can be repeated to treat new metastases in other regions of the brain without significantly increasing adverse effects.^54^ It is also suitable for patients with BMs that are not surgically accessible or who are not surgically fit. ASCO-SNO-ASTRO guidelines recommend SRS alone for one to four unresected BMs and postoperatively for patients who have undergone resection of one or two BMs.^55^ However, SRS use has expanded beyond its traditional application for patients with four or fewer BMs. The median OS after SRS varies, with 13.9 months for single BMs, 10.8 months for two to four metastases, and 7.5 months for five to 10 metastases.^56,57^ Some studies even suggest that patients with more than 10-15 BMs can be treated with SRS alone.^58,59^ These findings suggest that SRS may be a viable option for selected patients with up to 10 BMs, thus expanding its applicability in this patient population.

SRS has certain limitations compared with WBRT or surgery. SRS may be less effective in treating brain lesions >3 cm in diameter because of the need to limit the dose to surrounding normal brain tissue. In addition, it may take weeks to months for some lesions to respond or shrink after SRS, potentially delaying relief or symptoms caused by the tumor mass effect. Acute complications include nausea, vomiting, headache, and short-term cognitive deficits.^60^ Radiation necrosis is a chronic complication in 10% of patients treated with SRS; in about one third of these patients, necrosis is symptomatic, causing edema or decreased quality of life.^61,62^ Finally, unlike surgery, SRS does not enable pathologic diagnosis/confirmation, and unlike WBRT, SRS potentially could leave micrometastases untreated.

#### SRS After Surgical Resection

In a retrospective analysis,^63^ surgical resection followed by SRS yielded a substantial reduction in local recurrence compared with SRS as a stand-alone treatment, particularly in patients with BMs ≥2 cm (1-year local recurrence rate, 36.7% *v* 20.5%; *P* = .007). Moreover, the combination of surgery and SRS yielded a higher 2-year OS rate (38.9% *v* 19.8%; *P* = .01).

#### SRS Followed by WBRT

Although the focal nature of SRS was previously considered an advantage over WBRT, studies have shown that patients treated with SRS alone had higher rates of new BM development in areas outside the radiation field and of progression of treated lesions compared with patients treated with SRS followed by WBRT. In one study, patients treated with SRS combined with WBRT (n = 95) had intracranial tumor control rates of 93.7%, compared with 75.3 in those treated with SRS alone (n = 105; 95% CI, 7.8 to 29.0]; *P* < .001). According to another study,^64^ patients treated with only SRS had shorter median OS (7.5 months) and a higher recurrence rate at 12 months (76.4%) than did patients treated with SRS plus WBRT (OS, 8 months; recurrence rate, 48.8%).

In the randomized RTOG 9508 trial,^65^ which involved patients with one to three BMs, those who received SRS in addition to WBRT had a median OS of 6.5 months, compared with 4.9 months for those treated with WBRT only (*P* = .0393); In addition, the local recurrence or progression rate at 1 year was 29% in patients receiving WBRT only compared with 18% in those treated with WBRT plus SRS. These findings highlight the potential benefits of combining SRS with WBRT in managing oligometastatic lesions and particularly solitary BMs.

#### Postradiotherapy Neurocognitive Effects

The use of SRS alone did not result in worse neurologic function of the treated lesions versus SRS plus WBRT in patients with BMs from breast cancer.^66^ Neurologic function scores, assessed using the mini-mental scale, were 27.0 at a 30.5-month follow-up for WBRT plus SRS and 28.0 at 20.7 months for SRS alone. Therefore, the authors concluded that SRS could be used without WBRT, if periodic imaging of the brain is conducted.

A key toxic effect of WBRT for BMs is neurocognitive decline, despite attempted interventions to ameliorate it. Research has highlighted the role of hippocampal dysfunction in WBRT-induced cognitive decline.^67^ Verbal learning and memory, executive function, and verbal fluency are affected in WBRT, with potential long-term effects, whereas in SRS, short-term follow-up (1-4 months) commonly shows affected verbal learning and memory, fine motor coordination, and executive function, but these typically recover close to baseline cognitive performance over the long term.^68^

Memantine, an N-methyl-d-aspartate (NMDA) receptor antagonist, mitigates NMDA receptor stimulation, offering benefits in vascular dementia^69^ and neuroprotection in preclinical animal models of brain irradiation.^70^ RTOG 0614,^69^ a double-blind, placebo-controlled phase III trial, explored the use of memantine in preventing cognitive dysfunction in patients with BMs undergoing WBRT. In this study involving 508 patients, the memantine arm showed a cognitive failure rate of 53.8% compared with 64.9% in the placebo arm, suggesting potential effectiveness in mitigating cognitive decline during brain irradiation.

Studies such as RTOG 0933,^71^ using standardized cognitive assessments, have shown that hippocampal-sparing techniques such as intensity-modulated radiotherapy/shielding can significantly reduce cognitive decline compared with conventional WBRT. The primary end point of neurocognitive function showed a 4 month significant decline in only 7% of patients compared with 30% in historical controls. Moreover, a phase III NRG study^72^ indicated that WBRT with hippocampal shielding and memantine, compared with standard WBRT with memantine, did not adversely affect progression-free survival (PFS) or OS. In addition, there was no significant difference in grade ≥3 toxicity. Consequently, for patients with a good performance status and without hippocampal metastasis, WBRT with hippocampal shielding plus memantine may be a viable consideration.

In a randomized controlled trial for patients with resected BMs, patients receiving SRS experienced a longer period of cognitive deterioration–free survival (3.7 months [95% CI, 3.45 to 5.06]) compared with those receiving WBRT (3.0 months [95% CI, 2.86 to 3.25]).^73^ Another study showed that there were no discernible differences in OS (15.6 months for both) between SRS and WBRT.^74^ From these two studies, SRS showed a comparable efficacy with WBRT, while also carrying a reduced risk of cognitive decline. Consequently, when feasible, the preferred option after surgical resection is SRS.

In patients with one to three BMs, SRS alone was associated with lower cognitive dysfunction at 3 months compared with WBRT plus SRS (63.5% *v* 91.7%, *P* < .001).^75^ Among long-term survivors, cognitive deterioration occurred less frequently with SRS only at 3 months (45.5% *v* 94.1%, *P* = .007) and 12 months (60% *v* 94.4%, *P* = .04). These results suggest that SRS can be considered the standard of care for patients with one to three BMs as well. There was no significant difference in median OS between patients with two to four BMs and those with five to 10 BMs. These findings support the use of SRS as a treatment option for up to 10 BMs because of its minimally invasive technique and fewer side effects.^76^

## SYSTEMIC TREATMENT OF BREAST CANCER BMS

Temozolomide, an alkylating agent known for its effective penetration through the blood brain barrier (BBB), has been studied in the context of BMs. Several phase II studies^77-79^ have indicated improved response rates when temozolomide is combined with radiotherapy. However, none of these studies showed a significant improvement in OS. A phase I trial^80^ investigated combination therapy with temozolomide and capecitabine for breast cancer BMs and found that this treatment combination was well-tolerated and demonstrated significant antitumor activity, with an 18% objective response rate in the brain. However, OS was not addressed in this trial.

In a phase II trial,^81^ the combination of temozolomide with cisplatin demonstrated synergistic effects. Over 30% of patients with BMs from breast cancer experienced improvement after receiving this combination treatment, whereas another 16% maintained stable condition. These findings suggest the potential for additional combination therapies including targeted agents, antibody-drug conjugates, and temozolomide.

### HER2-Positive Disease

Metastatic tumor cells may find refuge in the brain in patients with HER2-positive breast cancer undergoing trastuzumab-based treatment.^27,67^ Trastuzumab exhibits low, likely nontherapeutic, levels in the CSF after intravenous administration; however, trastuzumab's penetration into the CSF is enhanced by impaired BBB conditions, such as meningeal carcinomatosis or disruption by radiotherapy. These findings support maintaining trastuzumab therapy in patients with BMs and extracranial responsive metastases.^9,82^

Currently, multiple studies of systemic therapy for HER2 positive BCBMs has been published (Table 2). Trastuzumab monotherapy showed survival benefits,^83^ as did trastuzumab plus pertuzumab.^84^ Changing systemic therapy after detection of oligometastatic (one to three) BMs prolonged OS and extracranial PFS, but not significantly.^85^ Thus, more investigation is needed for the appropriate systemic therapy in such cases.

**TABLE 2.** Current Published Clinical Studies of Treatment for Brain Metastases in Patients With HER2-Positive Breast Cancer

Study Name	First Author, Reference Citation	Intervention and Patients	Outcomes
Trastuzumab treatment beyond brain progression	Park et al^83^	Trastuzumab maintenance therapy in patients with BMs and extracranial responsive metastases	Significantly longer median OS (13.6 months [95% CI, 9.0 to 18.2]) compared with those without trastuzumab (5.5 months [95% CI, 0.0 to 13.6])
Dual HER2 blockade for breast cancer brain metastases	Bergen et al^84^	Trastuzumab + pertuzumab *v* other systemic therapies as first-line treatment after BM diagnosis	Significantly longer OS (44 months) compared with other HER2-targeted therapies, including trastuzumab alone, trastuzumab + lapatinib, lapatinib alone, T-DM1 (17 months), or no HER2-targeted therapy (3 months; *P* < .001)
Changing systemic therapy in relapsed breast cancer with BMs	Alhalabi et al^85^	Systemic therapy (not specified) changed when applicable per treating physician's discretion for patients with 1-3 BMs	Longer median OS (20.1 *v* 15.1 months) and extracranial PFS (14.9 *v* 11.6 months) in patients treated with changing systemic therapy
T-DM1 activity in HER2-positive breast cancer brain metastases	Bartsch et al^86^	T-DM1 *v* physician's choice	Median OS improvement with T-DM1 vs physician's choice in patients with baseline BMs (17.3 *v* 12.6 months)
TH3RESA trial comparing T-DM1 with physician's choice	Krop et al^87^	T-DM1 *v* physician's choice (subgroup analysis of patients with *v* without BM)	No statistically significant difference in OS between T-DM1 and physician's choice (95% CI, not provided)
T-DM1 and brain metastases in a real-world study	Fabi et al^135^	T-DM1	Median PFS and OS of 7 and 14 months in patients with BMs. OR in 24.5% (N = 13), with CR in 3.8% (N = 2) and stable disease in 30.1% (N = 16)
Lapatinib access into normal brain and metastases	Saleem et al^89^	Radiolabeled lapatinib and PET scans before and after oral lapatinib (8 days) in patients with or without 1 or more 1-cm BM	PET demonstrated lapatinib's ability to penetrate BBB and shrink HER2-positive BMs
Lapatinib in patients with brain metastases	Lin et al^90^	Lapatinib in patients with CNS progression after previous trastuzumab and cranial radiotherapy	Objective CNS response in 20% of patients
Lapatinib plus capecitabine in patients with previously untreated brain metastases from HER2-positive metastatic breast cancer (LANDSCAPE)	Bachelot et al^91^	Lapatinib + capecitabine for previously untreated BMs	Patients with >50% reduction of CNS volume had longer median PFS than patients with <50% reduction (3.38 *v* 2.07 months)
CEREBEL study (EGF111438)	Pivot et al^136^	Trastuzumab + capecitabine *v* lapatinib + capecitabine in patients without baseline CNS metastases	No difference in the incidence of BMs between the two treatment groups
NEfERT-T trial	Awada et al^93^	Neratinib + trastuzumab *v* trastuzumab + paclitaxel	Comparable PFS between the two treatment groups, but symptomatic or progressive CNS recurrences were more frequent in the trastuzumab + paclitaxel group (17% *v* 8%, *P* = .002)
Phase III NALA trial	Saura et al^94^	Neratinib + capecitabine *v* lapatinib + capecitabine in patients with metastatic disease previously treated with ≥2 HER2-directed regimens	Superior PFS (HR, 0.76 [95% CI, 0.63 to 0.93]; *P* = .0059) and fewer interventions for CNS disease (cumulative incidence, 22.8% *v* 29.2%; *P* = .043) occurred with neratinib + capecitabine
Phase III ExteNET trial, Efficacy of neratinib in early-stage HER2-positive breast cancer	Holmes et al^95^	Neratinib *v* placebo 1 year after definitive primary surgery in women with early-stage disease who had completed neoadjuvant or adjuvant trastuzumab + chemotherapy	Improved 5-year distant disease-free survival of 7% and 10-year OS of 9.1% with neratinib in patients without pathologic CR to neoadjuvant treatment
Phase Ib study of tucatinib and T-DM1 in *ERBB2*-positive breast cancer	Borges et al^96^	Tucatinib + T-DM1 in patients with previously treated ERBB2/HER2-positive metastatic breast cancer, both with and without BMs	Acceptable toxicity and signs of antitumor activity. Median PFS of 6.7 months (95% CI, 4.1 to 10.2) and OR duration of 6.9 months (95% CI, 1.45 to 19.48) in patients with BMs
HER2CLIMB study	Murthy et al^97^	Tucatinib + capecitabine + trastuzumab	42% of patients with BMs achieved brain-specific OR
HER2CLIMB study comparing tucatinib with placebo	Murthy et al^98^	Tucatinib + capecitabine + trastuzumab *v* placebo + capecitabine + trastuzumab (subgroup analysis of patients with BMs at baseline)	Among patients with baseline BMs, those in the tucatinib combination group, vs the placebo combination group, had better median PFS (7.6 *v* 5.4 months), OS (HR, 0.58 [95% CI, 0.40 to 0.85]), and PFS (HR, 0.48 [95% CI, 0.34 to 0.69])
Trastuzumab deruxtecan in HER2-low advanced breast cancer	Modi et al^137^	Trastuzumab deruxtecan (DS8201)	PFS for patients with stable BMs was 18.1 months, and OS was not reached

Abbreviations: BBB, blood brain barrier; BM, brain metastasis; CR, complete response; HER2, human epidermal growth factor receptor-2; HR, hazard ratio; OR, overall response; OS, overall survival; PET, positron emission tomography; PFS, progression-free survival; T-DM1, ado-trastuzumab emtansine.

Ado-trastuzumab emtansine (T-DM1), an antibody-drug conjugate, mitigates the cytotoxic effects of DM1 by binding it to trastuzumab. However, because of its high molecular weight, T-DM1 has difficulty in crossing the BBB except when the BBB is disrupted.^86^ Among patients with BMs in the TH3RESA study, T-DM1 prolonged OS, but not significantly compared with trastuzumab- or lapatinib-containing therapy with more than two previous lines of therapy.^87^ A retrospective study showed that T-DM1 revealed an overall response in 13 (24.5%) of 53 patients, indicating efficacy in controlling BMs.^88^

The KATHERINE trial of postneoadjuvant anti-HER2 treatment for patients without complete pathologic response found that BMs occurred as the first metastatic site of 5.9% of patients treated with T-DM1 and 4.3% of patients treated with trastuzumab.^27^

Lapatinib, a small-molecule tyrosine kinase inhibitor of the EGFR and HER2 pathways, can penetrate the BBB. Previous studies^40,89^ elicit an objective CNS response^90^ and prolong PFS.^91^ However, the CEREBEL study comparing trastuzumab-capecitabine and lapatinib-capecitabine detected no difference in the incidence of BMs.^92^ The LANDSCAPE trial, a phase 2 study with a single group, demonstrated an approximately 66% objective CNS volumetric response rate among 44 patients receiving combination lapatinib and capecitabine as their initial therapy for BCBMs.^91^

A phase II trial comparing neratinib, a tyrosine kinase inhibitor targeting EGFR, HER2, and HER4, combined with paclitaxel versus trastuzumab-paclitaxel found no difference in PFS.^93^ However, neratinib-capecitabine yielded PFS benefits,^94^ and the phase III ExteNet trial^95^ showed that neratinib after primary surgery yielded survival benefits and fewer CNS events.

Tucatinib (a tyrosine kinase inhibitor of the HER2 gene *ERBB2*) plus T-DM1 demonstrated acceptable toxicity and antitumor activity in a phase Ib study.^96^ In the phase Ib HER2CLIMB study, tucatinib-capecitabine-trastuzumab yielded brain-specific objective responses^97^ and survival benefits in patients with BMs at baseline.^98^

Trastuzumab deruxtecan (DS8201), an antibody-drug conjugate with targeted antitumor activity, may offer survival benefits to patients with stable BMs.^99^

### HR-Positive/HER2-Negative Disease

Endocrine therapies such as tamoxifen, fulvestrant, and aromatase inhibitors have been used consistently to treat breast cancer because of their low toxicity profile,^100-102^ and the demonstrable benefit of adding CDK4/6 inhibitors such as abemaciclib, ribociclib, and palbociclib was evident. Abemaciclib has demonstrated greater ability than other CDK4/6 inhibitors to reach effective levels in brain metastatic tissue.^103^ By contrast, a clinical study^104^ involving postmenopausal women showed that the estrogen receptor antagonist elacestrant could penetrate the BBB. Table 3 shows ongoing trials in this subtype and others, including TNBC.

**TABLE 3.** Ongoing Clinical Trials Focused on Breast Cancer Brain Metastasis Including Various Subpopulations

NCT Identifier	Phase	Treatment	Biomarkers
All subtypes			
NCT03807765	I	SBRT + nivolumab	All subtypes
NCT03449238	I/II	SRS + pembrolizumab	All subtypes
NCT03697343	III	FSRT *v* comparison with single session radiosurgery in patients with larger brain metastases (2-4 cm)	All subtypes
NCT05703269	III	SSRS *v* FSRS	All subtypes
NCT03075072	III	Hippocampal sparing WBRT *v* SRS with 5-20 BMs	All subtypes
NCT04899908	II	SRS ± AGuIX gadolinium-based nanoparticles	All subtypes
NCT05222620	II	SRS *v* FSRS—FRACTIONATE trial	All subtypes
NCT03550391	III	SRS *v* HA-WBRT plus memantine for ≥5 more BMS	All subtypes
NCT04030507	II	Preventive: Screening MRI of the brain in MBCs	All subtypes
NCT05115474	II	Screening brain MRIs in stage IV breast cancer	All subtypes
NCT04420598	II	T-DXd	All subtypes
NCT03994796	II	Genetic testing in guiding treatment for patients with BMs	All subtypes
HR+ HER2–			
NCT04791384	Ib/II	Elacestrant and abemaciclib	HR+/HER2–
NCT05293964	I	SCR-6852, palbociclib	HR+/HER2–
HER2+			
NCT03933982	II	Pyrotinib + vinorelbine	HER2+
NCT04639271	II	Pyrotinib + trastuzumab + Nab paclitaxel	HER2+
NCT05042791	II	Concomitant SBRT pyrotinib + capecitabine	HER2+
NCT01494662	II	Preoperative neratinib with or without capecitabine or T-DM1	HER2+
NCT04760431	II	THP *v* TH + TKI (neratinib or tucatinib; HER2BRAIN)	HER2+
NCT05323955	II	HP or T-DM1 + tucatinib	HER2+
NCT05593094	I	ZN-A-1041 or ZN-A-1041 combination	HER2+
NCT04512261	II	Tucatinib + trastuzumab + pembrolizumab (TOPAZ)	HER2+
NCT04739761	III	T-DXd	HER2+
NCT04760431	II	THP *v* TH-pyrotinib	HER2+
NCT04509596	I	DZD1516 with capecitabine or T-DM1	HER2+
NCT05018702	II	ARX788	HER2+
NCT04539938	II	T-DXd, tucatinib	HER2+
NCT03190967	I/II	Metronomic temozolomide and T-DM1	HER2+
NCT03765983	II	Paxalisib (GDC-0084) + trastuzumab	HER2+
NCT04348747	II	Anti-HER2/HER3 dendritic cell vaccine ID, celecoxib, interferon alfa-2b followed by pembrolizumab	HER2+
NCT03714243	NA	HIFU (ExAblate BBBD)	HER2+
NCT04582968	I/II	SRS or WBRT and pyrotinib + capecitabine	HER2+
NCT04158947	II	Afatinib, T-DM1	HER2+
HER2–			
NCT03328884	II	MM-39 (phenomenal)	HER2–
NCT04965064	II	Pyrotinib, capecitabine	HER2–
NCT04647916	II	Sacituzumab govitecan	HER2–
NCT04923542	II	SRS + abemaciclib/ET	HER2–
NCT01770353	I	MM-398 (nanoliposomal irinotecan)	HER2–
TNBC			
NCT04348747	II	Anti-HER2/HER3 dendritic cell vaccine ID, celecoxib, interferon alfa-2b followed by pembrolizumab	TNBC
NCT03995706	I	Sacituzumab govitecan	TNBC
NCT02574455	III	ASCENT study, sacituzumab govitecan	TNBC
NCT05255666	II	Nal-IRI, pembrolizumab	TNBC
NCT03483012	II	SBRT + atezolizumab	TNBC
NCT04434560	II	Nivolumab + ipilimumab	TNBC
NCT03483012	II	Atezolizumab + stereotactic radiation	TNBC
NCT03761914	I/II	Galinpepimut-S + pembrolizumab	TNBC
NCT04303988	II	SHR-1316 + bevacizumab + cisplatin/carboplatin	TNBC
NCT04789668	I/II	Bintrafusp alfa + pimasertib	TNBC
NCT05305365	II	QBS72S	TNBC
NCT04711824	I/II	Olaparib + SRS → pembrolizumab	TNBC or BRCA-mutated BC

Abbreviations: ADC, antibody-drug conjugate; BC, breast cancer; Chemo, chemotherapy; ET, endocrine therapy; FSRS, fractionated stereotactic radiosurgery; FSRT, fractionated stereotactic radiotherapy; HA-WBRT, hippocampal-avoidant whole-brain radiotherapy; HER2, human epidermal growth factor receptor 2; HIFU, high-intensity focused ultrasound; HR, hormone receptor; HS-WBRT, hippocampal-sparing whole-brain radiation therapy; IO, immunotherapy; ID, intradermally; MRI, magnetic resonance imaging; NCT, national clinical trial; PARPi, poly(ADP-ribose) polymerase inhibitor; RT, radiation therapy; SRS, stereotactic radiotherapy; SSRS, single-fraction stereotactic radiosurgery; T-DM1, trastuzumab emtansine; T-DXd, trastuzumab deruxtecan; TH, trastuzumab and pertuzumab; THP, trastuzumab, pertuzumab, and docetaxel; TKI, tyrosine kinase inhibitor; TNBC, triple-negative breast cancer; WBRT, whole-brain radiation therapy.

### TNBC

Despite limited data availability, ongoing clinical studies aim to improve treatment strategies for TNBC. These treatments include chemotherapy with capecitabine,^105^ platinum-based agents,^106^ and topoisomerase inhibitors such as etirinotecan pegol (NKTR-102).^107^ A phase II study is evaluating safety, objective response rate, and systemic and CNS-specific PFS in patients with CNS disease, including those with leptomeningeal disease (LMD), treated with pembrolizumab.^108^

The published literature underscores the need for clinical trials involving poly(ADP-ribose) polymerase inhibitors, including niraparib, olaparib, talazoparib, and veliparib (ClinicalTrials.gov identifier: NCT02595905), or alternative antibreast cancer medications that can penetrate the CNS to treat BMs in patients with breast cancer with germline BRCA mutations. This imperative is particularly crucial for individuals with recurrent TNBC,^11^ including those with and without *gBRCA* mutations.

### LMD

The prognosis for patients with LMD is poor. Various prognostic indices had been developed that may help in the clinical decision making process of predicting survival.^109-111^

Furthermore, patterns of LMD involvement and response to therapy may be of clinical significance and value for future research development.^112,113^ The standard of care results in a 1 year OS rate of 13% of patients yet the median OS is 3-4 months after treatment.^114-117^ However, OS can vary among biologic subtypes. The median OS is 4.4 months for patients with HER2-positive breast cancer and LMD, 3.7 months for patients with HR-positive/HER2-negative breast cancer and LMD, and 2.2 months for those with TNBC and LMD.^115^ Some single-institution studies^116,117^ found that older age at the time of TNBC LMD diagnosis, the presence of previous or concomitant BMs, and a low albumin level were all indicators of a less favorable prognosis. According to the 2022 National Comprehensive Cancer Network guidelines, individuals with favorable characteristics (KPS ≥60, absence of significant neurologic impairments, limited systemic disease, and viable systemic treatment alternatives) who are diagnosed with LMD are recommended to undergo SRS or involved-field radiotherapy (IFRT) to treat symptomatic bulky disease.^118-120^ Methotrexate and thiotepa,^121^ liposomal cytarabine,^122^ and topotecan^123^ have been investigated for intrathecal administration with overall response rates of up to 55%. The median OS for patients receiving these intrathecal drugs typically ranges from 4 to 5 months.^124,125^ Response is evaluated using the Leptomeningeal Assessment in Neuro-Oncology (LANO) scorecard.^126^ Concomitant administration of radiotherapy and intrathecal chemotherapy is generally not recommended because of the high incidence of neurotoxicities such as acute cerebral meningitis, chronic encephalopathy, and leukoencephalopathy; severe CNS toxicities are reported in 2%-27% of patients.^127^

In cases where patients have unfavorable features, such as substantial CNS disease, the option of palliative IFRT may be considered.^120^ In a randomized phase II trial^128^ comparing proton craniospinal irradiation (pCSI) with IFRT in patients with LMD, the median CNS PFS was 7.5 months for pCSI and 2.3 months for IFRT. In addition, the trial demonstrated an OS advantage of 9.9 months for pCSI versus 6 months for IFRT, with no discernible disparity in toxicities. The goal of high-dose intravenous and intrathecal (IT; via lumbar puncture or ventricular Ommaya reservoir) chemotherapy is to increase the concentration of antineoplastic drugs within the CNS at effective doses.^67^ Drugs delivered through the Ommaya reservoir had double the effect on OS (9.2 months) compared with IT delivery (4 months).^129^ Some small studies have shown that high-dose chemotherapy, such as methotrexate, can achieve a CNS overall response rate of approximately 30%.^130,131^ Nevertheless, the potential toxicity associated with these treatments must be carefully evaluated in relation to a patient's performance status and existing medical conditions. Limited data are available on the use of combined high-dose intravenous and intrathecal chemotherapy.

ANG1005 (paclitaxel trevatide), an investigational peptide-drug conjugate, has good efficacy and antitumor CNS activity.^132^ An ongoing phase III study, ANGLeD, is investigating the treatment effect of ANG1005 versus chemotherapy in patients with HER2-negative breast cancer with BMs and newly diagnosed LMD (ClinicalTrials.gov identifier: NCT03613181). Table 4 shows SRS-based trials, and Table 5 depicts trials specifically for patients with LMD.

**TABLE 4.** SRS-Focused Clinical Trials in Breast Cancer Brain Metastases

NCT Identifier	Phase	Treatment	Population
NCT03807765	I	SBRT + nivolumab	All subtypes
NCT03449238	I/II	SRS + pembrolizumab	All subtypes
NCT03697343	III	FSRT *v* SSRS for BMs (2-4 cm)	All subtypes
NCT05703269	III	SSRS *v* FSRS	All subtypes
NCT03075072	III	Hippocampal-sparing WBRT *v* SRS with 5-20 BMs	All subtypes
NCT04899908	II	SRS ± AGuIX gadolinium-based nanoparticles	All subtypes
NCT05222620	II	SRS compared with FSRS—FRACTIONATE trial	All subtypes
NCT03550391	III	SRS *v* HA-WBRT plus memantine for ≥5 more BMs	All subtypes
NCT04582968	I/II	SRS or WBRT and pyrotinib + capecitabine	HER2+
NCT04923542	II	SRS + abemaciclib/ET	HER2–
NCT03483012	II	SBRT + atezolizumab	TNBC
NCT03483012	II	Atezolizumab + SRS	TNBC
NCT04711824	I/II	Olaparib + SRS → pembrolizumab	TNBC or BRCA-mutated BC

Abbreviations: AGuIX, activation and guidance of irradiation by X-ray; BC, breast cancer; BM, brain metastases; ET, endocrine therapy; FSRS, fractionated stereotactic radiosurgery; FSRT, fractionated stereotactic radiotherapy; HA-WBRT, hippocampal-avoidant whole-brain radiotherapy; HER2, human epidermal growth factor receptor 2; NA, not applicable; NCT, national clinical trial; SRS, stereotactic radiotherapy; SSRS, single-fraction stereotactic radiosurgery; TNBC, triple-negative breast cancer; WBRT, whole-brain radiation therapy.

**TABLE 5.** LMD-Focused Clinical Trials in Breast Cancer Brain Metastases

NCT Identifier	Phase	Treatment	Population
NCT03696030	I	HER2-CAR T cells	HER2+
NCT03613181	III	ANG1005 in LMD	HER2–
NCT02422641	I	Prospective evaluation of high-dose systemic methotrexate	All subtypes
NCT05746325	NA	Tumor treating fields for the treatment of spinal LMD	All subtypes

Abbreviations: CAR, chimeric antigen receptor T-cell therapy; HER2, human epidermal growth factor receptor-2; LMD, leptomeningeal disease; NA, not applicable; NCT, national clinical trial.

## PREVENTION OF BRAIN METASTASIS

The prevention of primary and secondary BMs in breast cancer is an evolving field, incorporating immunotherapy, precision medicine, and therapeutic combinations to advance patient care and outcomes.

In the NEfERT-T trial,^93^ symptomatic or progressive CNS recurrences were observed in 20 patients (8.3%) treated with the neratinib-paclitaxel group, compared with 41 patients (17.3%) in the trastuzumab-paclitaxel group (relative risk, 0.48 [95% CI, 0.29 to 0.79]; *P* = .002). In the NALA trial,^94^ BM recurrence occurred in a lower proportion of patients in the neratinib-capecitabine group compared with those in the lapatinib-capecitabine group (cumulative incidence of intervention, 22.8% *v* 29.2%, respectively). These data serve as a proof of concept and a basis of hypothesis generation that neratinib may serve as a CNS metastasis prevention agent.

The Cleveland Clinic is leveraging a vaccine strategy as a potential preventive measure against BMs (ClinicalTrials.gov identifier: NCT04674306). The initiative underscores the importance of immunotherapy in breast cancer prevention

In the context of early-stage HER2-positive breast cancer with residual disease after neoadjuvant therapy, the A011801 (CompassHER2 RD) trial is investigating BM-free survival with postneoadjuvant T-DM1 + tucatinib or T-DM1 + placebo.^133^ For patients with metastatic HER2-positive disease experiencing isolated intracranial progression on trastuzumab/pertuzumab or T-DM1, the BRIDGET trial aims to prevent secondary BMs by incorporating tucatinib (ClinicalTrials.gov identifier: NCT05323955) and a phase I trial^134^ will assess low-dose temozolomide and T-DM1 for prevention of secondary prevention of breast cancer brain metastasis.

In conclusion, BMs continue to pose a significant clinical challenge, prompting the need for further research. Preclinical and clinical studies are providing valuable insights into the biology of BMs and the role of the microenvironment. The use of hippocampal shielding and memantine helped abrogate neurocognitive dysfunction, but improvement is needed. Furthermore, advancements in systemic therapies that can penetrate the BBB or in manipulating the microenvironment may hold promise for improving treatment outcomes.
